# The impact of long-term adherence to guideline-directed medical therapy on outcomes in peripheral artery disease

**DOI:** 10.1177/1358863X251410939

**Published:** 2026-02-09

**Authors:** Tjaša Furlan, Janez Bijec, Petra Došenović Bonča, Irena Ograjenšek, Borut Jug

**Affiliations:** 1 Medical Faculty, University of Ljubljana, Ljubljana, Slovenia; 2 General Hospital Trbovlje, Trbovlje, Slovenia; 3 Faculty of Electrical Engineering, University of Ljubljana, Ljubljana, Slovenia; 4 School of Economics and Business, University of Ljubljana, Ljubljana, Slovenia; 5 Department of Vascular Diseases, University Medical Centre Ljubljana, Ljubljana, Slovenia

**Keywords:** adherence, mortality, peripheral artery disease (PAD), practice guidelines, rehospitalization

Peripheral artery disease (PAD) is a prevalent manifestation of systemic atherosclerosis associated with high cardiovascular risk.^1^ Guideline-directed medical therapy (GDMT), including antiplatelet agents, lipid-lowering therapy, and renin–angiotensin system inhibitors (RAASi), reduces adverse outcomes,^2,3^ yet uptake and adherence in PAD remain suboptimal compared with other atherosclerotic conditions, such as coronary or cerebrovascular disease.^4,5^ Therapy initiation at discharge has been studied,^4–6^ yet the long-term prognostic significance of sustained adherence in PAD populations remains insufficiently characterized.

We conducted a nationwide retrospective cohort study including all patients hospitalized with PAD in Slovenia between January 1, 2015 and June 30, 2021, with a median follow-up of 34.5 (IQR: 16.8–53.8) months. Data were linked across the Hospital Discharge Statistics Database, the National Mortality Registry, and the Medication Reimbursement Database. PAD diagnoses were identified using International Classification of Diseases, Tenth Revision (ICD-10) code I70.x. Adherence was assessed from pharmacy dispensation data using a continuous measure of adherence (CMA7),^7^ calculated within rolling 120-day windows, with overlap and carry-over of surplus medication.^7^ Adherence was modeled as a time-varying covariate, both continuously and dichotomized at ⩾ 80%.^8^ Each CMA was divided by 10 so that a one-unit change in the hazard ratio (HR) corresponded to a 10% absolute change in adherence. In addition to class-specific adherence, we created composite GDMT adherence measures: (i) an ordinal count (zero to three classes adherent at CMA7 ⩾ 80%) across antiplatelet, lipid-lowering, and RAASi therapy; and (ii) a binary composite (adherent to all three vs fewer than three). The primary outcome was a composite of all-cause mortality or hospitalization for nonfatal myocardial infarction, ischemic stroke, or PAD-related events. Associations were analyzed using multivariable Cox regression with time-dependent covariates, adjusted for age, sex, comorbidities, disease severity, and socioeconomic status. The National Medical Ethics Committee approved the study (KME-0120-29/2022/6). Given the retrospective, registry-based study design, the requirement for informed consent was waived.

A total of 8811 patients were included. Within 120 days of discharge, only 4191 (47.6%) patients filled at least one prescription for all three GDMT classes (median age 69 years, 38% women). Baseline use was highest for antiplatelet therapy (84%), followed by lipid-lowering therapy (66%) and angiotensin-converting enzyme inhibitors or angiotensin receptor blockers (ACEi/ARBs) (64%). Adherence declined over time across classes, most prominently for lipid-lowering therapy (Supplemental Figure). Predictors of poor adherence included chronic limb-threatening ischemia (CLTI), atrial fibrillation with anticoagulation, cancer, and lower socioeconomic status, whereas diabetes, hypertension, and use of other antihypertensive drugs were associated with greater adherence. Higher adherence was associated with significantly improved outcomes (Table 1). Each 10% increase in adherence was independently associated with a lower risk of the composite endpoint: HR 0.96 (95% CI 0.93–0.98; *p* = 0.002) for lipid-lowering therapy, HR 0.95 (95% CI 0.92–0.99; *p* = 0.009) for antiplatelet therapy, and HR 0.97 (95% CI 0.95–1.00; *p* = 0.005) for ACEi/ARBs. These associations were consistent when adherence was analyzed dichotomously (⩾ 80%) and when modeled using penalized splines. In composite analyses, the ordinal zero to three class score showed a significant linear trend (orthogonal polynomial contrast) toward lower risk with increasing adherence (HR 0.74, 95% CI 0.66–0.83; *p* < 0.001). For the binary composite, patients with fewer than three adherent classes had higher risk than those adherent to all three (HR 1.40, 95% CI 1.23–1.58; *p* < 0.001); equivalently, adherence to all three classes was associated with a 29% lower hazard (HR 0.71, 95% CI 0.63–0.81).

**Table 1.** Predictors of survival in patients hospitalized with PAD (Cox models with continuous and dichotomized measures of adherence).

Predictors	Survival (CMA7)			Survival (low vs high adherence)		
	Estimates	95% CI	*p*	Estimates	95% CI	*p*
Lipid-lowering therapy adherence	0.96	0.93–0.98	**0.002**	0.75	0.62–0.90	**0.003**
RAASi adherence	0.97	0.95–1.00	**0.005**	0.82	0.69–0.98	**0.030**
Antiplatelet therapy adherence	0.95	0.92–0.99	**0.009**	0.70	0.57–0.85	**< 0.001**
DAPT adherence	0.99	0.96–1.02	0.576	0.99	0.95–1.02	0.559
Claudication	0.91	0.72–1.15	0.439	0.91	0.72–1.14	0.420
Other symptoms of PAD (rest pain, ulceration)	0.62	0.46–0.85	**0.004**	0.62	0.45–0.85	**0.003**
Age group: < 60 years	0.71	0.40–1.26	0.265	0.71	0.40–1.26	0.255
Age group: 60–69 years	0.96	0.56–1.67	0.903	0.96	0.55–1.65	0.876
Age group: 70–79 years	1.03	0.59–1.80	0.913	1.03	0.59–1.80	0.915
Age group: ⩾ 80	0.77	0.42–1.41	0.420	0.76	0.42–1.40	0.399
Male sex	1.22	1.02–1.47	**0.039**	1.23	1.02–1.48	**0.037**
Diabetes mellitus	1.14	0.92–1.41	0.260	1.14	0.92–1.41	0.258
Arterial hypertension	0.82	0.64–1.03	0.101	0.82	0.65–1.04	0.108
Ischemic heart disease	1.20	0.77–1.87	0.483	1.20	0.77–1.87	0.494
Heart failure	1.33	0.77–2.31	0.348	1.33	0.77–2.30	0.352
Atrial fibrillation	1.24	0.67–2.31	0.457	1.21	0.66–2.25	0.508
COPD/asthma	0.80	0.54–1.16	0.269	0.79	0.54–1.16	0.261
Dementia	1.09	0.43–2.76	0.868	1.12	0.44–2.84	0.828
Depression	1.24	0.85–1.81	0.299	1.22	0.84–1.79	0.326
Chronic kidney disease	1.03	0.68–1.54	0.901	1.03	0.69–1.54	0.898
Cancer	1.06	0.58–1.92	0.852	1.07	0.59–1.95	0.820
Number of diagnoses	1.03	0.97–1.10	0.357	1.03	0.96–1.10	0.362
Number of procedures	1.00	0.98–1.03	0.712	1.00	0.98–1.03	0.763
Revascularization	1.22	0.98–1.51	0.092	1.22	0.98–1.51	0.092
Anticoagulation	1.03	0.72–1.46	0.886	1.02	0.72–1.46	0.895
Beta blocker	0.71	0.55–0.92	**0.013**	0.71	0.55–0.92	**0.014**
Calcium channel blocker	1.20	0.96–1.51	0.112	1.21	0.96–1.52	0.109
Proton pump inhibitor	1.12	0.92–1.38	0.294	1.13	0.92–1.38	0.287
Diuretic	1.09	0.87–1.37	0.461	1.09	0.87–1.36	0.467
University hospital	1.18	0.92–1.50	0.189	1.18	0.92–1.51	0.183
Socio-economic status (below median income)	0.81	0.63–1.04	0.101	0.80	0.63–1.03	0.094

Bold *p* values are statistically significant.

CMA7, continuous measure of adherence; COPD, chronic obstructive pulmonary disease; DAPT, dual antiplatelet therapy; PAD, peripheral artery disease; RAASi, renin–angiotensin system inhibitors.

These findings emphasize two major challenges. First, initiation and persistence with GDMT in PAD remain suboptimal, even in Slovenia where patients face no out-of-pocket costs for these medications. This gap highlights barriers beyond affordability, including health literacy, multimorbidity, and system-level fragmentation. Second, the results underscore the importance of sustained adherence, with incremental benefits observed for each therapy class. The magnitude of association—3–5% lower event risk per 10% increase in adherence—parallels findings from coronary populations, and aligns with contemporary PAD guideline recommendations for comprehensive secondary prevention. Together with the composite analyses, these data support a graded, dose–response relationship between breadth of GDMT adherence and improved outcomes.

Our analysis extends beyond prior statin-focused adherence studies by concurrently evaluating all three GDMT classes, underscoring the additive value of comprehensive therapy. Identification of vulnerable subgroups, such as those with lower socioeconomic status, may inform targeted adherence interventions.

Limitations include the observational design, reliance on dispensation data (which may not fully reflect ingestion), and possible coding inaccuracies. Nevertheless, strengths include the nationwide scope, avoidance of primary nonadherence bias, time-varying adherence modeling, and complete capture of mortality and hospitalization outcomes.

In conclusion, sustained adherence to GDMT in PAD is independently associated with significantly reduced risk of death and major cardiovascular events, yet uptake and persistence remain inadequate. Composite adherence results (HR 0.74 linear trend across zero to three classes; HR 0.71 for all three vs fewer than three) reinforce a clinically meaningful dose–response. Closing this adherence gap represents a major opportunity to improve outcomes in this high-risk population.

## Supplemental Material

sj-docx-1-vmj-10.1177_1358863X251410939 – Supplemental material for The impact of long-term adherence to guideline-directed medical therapy on outcomes in peripheral artery disease Supplemental material, sj-docx-1-vmj-10.1177_1358863X251410939 for The impact of long-term adherence to guideline-directed medical therapy on outcomes in peripheral artery disease by Tjaša Furlan, Janez Bijec, Petra Došenović Bonča, Irena Ograjenšek and Borut Jug in Vascular Medicine
