# Supplementary material for: The impact of long-term adherence to guideline-directed medical therapy on outcomes in peripheral artery disease
Source: Vasc Med. 2026 Feb 9;31(2):216–8. doi: 10.1177/1358863X251410939 (PMC13109592; doi:10.1177/1358863X251410939)
Supplement: sj-docx-1-vmj-10.1177_1358863X251410939 – Supplemental material for The impact of long-term adherence to guideline-directed medical therapy on outcomes in peripheral artery disease [file sj-docx-1-vmj-10.1177_1358863X251410939.docx]

**Supplemental figure.** Time trends for continuous measures of adherence.


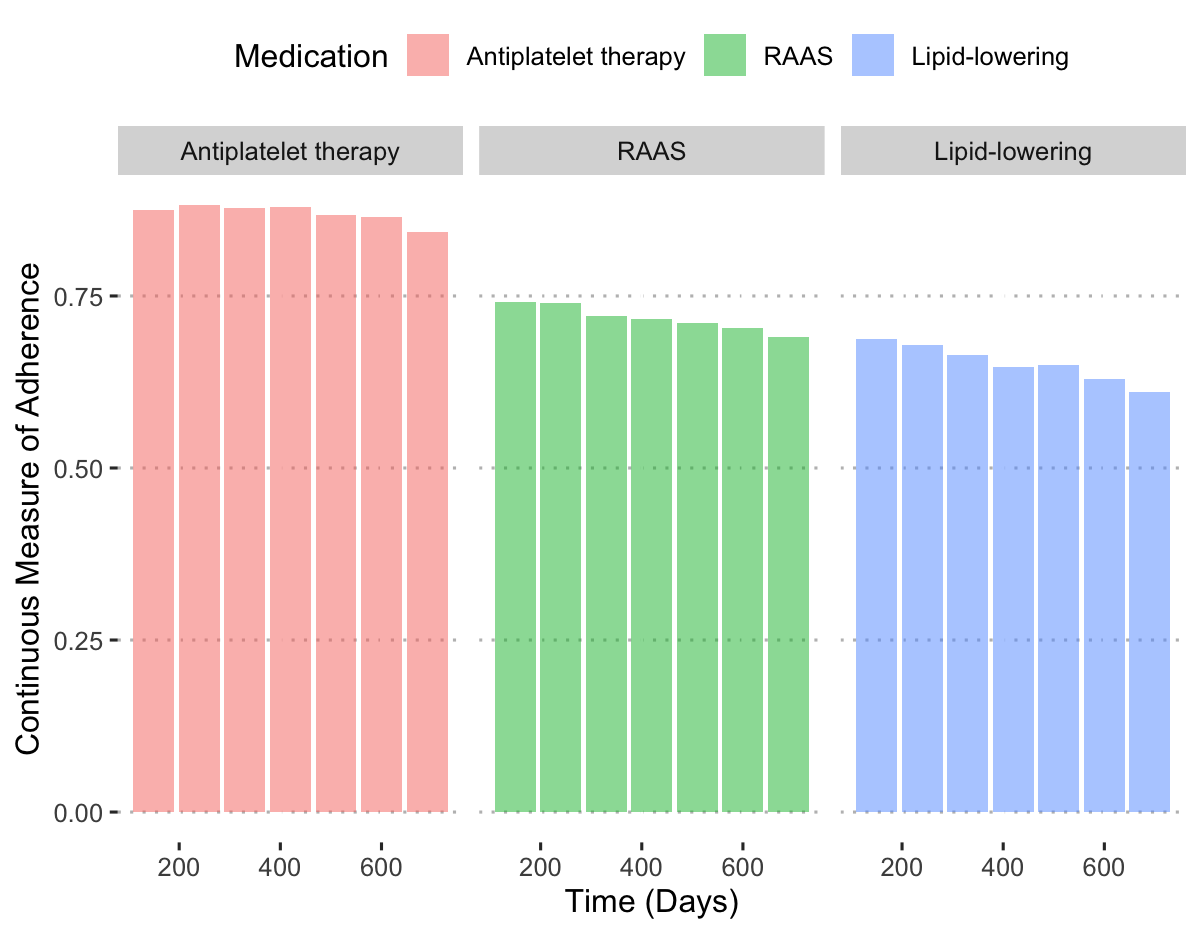


ACEi

/ARBs

ACEi/ARBs

ACEi/ARBs — angiotensin converting enzyme inhibitors or angiotensin receptor blockers
